# Multiple Sclerosis Identification by 14-Layer Convolutional Neural Network With Batch Normalization, Dropout, and Stochastic Pooling

**DOI:** 10.3389/fnins.2018.00818

**Published:** 2018-11-08

**Authors:** Shui-Hua Wang, Chaosheng Tang, Junding Sun, Jingyuan Yang, Chenxi Huang, Preetha Phillips, Yu-Dong Zhang

**Affiliations:** ^1^School of Computer Science and Technology, Henan Polytechnic University, Jiaozuo, China; ^2^School of Architecture Building and Civil Engineering, Loughborough University, Loughborough, United Kingdom; ^3^The Faculty of Computer Science and Engineering, Xi'an University of Technology, Xi'an, China; ^4^Department of Computer Science and Technology, Tongji University, Shanghai, China; ^5^West Virginia School of Osteopathic Medicine, Lewisburg, WV, United States; ^6^Department of Informatics, University of Leicester, Leicester, United Kingdom

**Keywords:** multiple sclerosis, deep learning, convolutional neural network, batch normalization, dropout, stochastic pooling

## Abstract

**Aim:** Multiple sclerosis is a severe brain and/or spinal cord disease. It may lead to a wide range of symptoms. Hence, the early diagnosis and treatment is quite important.

**Method:** This study proposed a 14-layer convolutional neural network, combined with three advanced techniques: batch normalization, dropout, and stochastic pooling. The output of the stochastic pooling was obtained via sampling from a multinomial distribution formed from the activations of each pooling region. In addition, we used data augmentation method to enhance the training set. In total 10 runs were implemented with the hold-out randomly set for each run.

**Results:** The results showed that our 14-layer CNN secured a sensitivity of 98.77 ± 0.35%, a specificity of 98.76 ± 0.58%, and an accuracy of 98.77 ± 0.39%.

**Conclusion:** Our results were compared with CNN using maximum pooling and average pooling. The comparison shows stochastic pooling gives better performance than other two pooling methods. Furthermore, we compared our proposed method with six state-of-the-art approaches, including five traditional artificial intelligence methods and one deep learning method. The comparison shows our method is superior to all other six state-of-the-art approaches.

## Introduction

Multiple sclerosis (abbreviated as MS) is a condition that affects the brain and/or spinal cord (Chavoshi Tarzjani et al., 2018). It will lead to a wide range of probable symptoms, likely with balance (Shiri et al., 2018), vision, movement, sensation (Demura et al., 2016), etc. It has two main types: (i) relapsing remitting MS and (ii) primary progressive MS. More than eight out of every ten diagnosed MS patients are of the “relapsing remitting” type (Guillamó et al., 2018).

MS diagnosis may be confused with other white matter diseases, such as neuromyelitis optica (NMO) (Lana-Peixoto et al., 2018), acute cerebral infarction (ACI) (Deguchi et al., 2018), acute disseminated encephalomyelitis (ADEM) (Desse et al., 2018), etc. Hence, accurate diagnosis of MS is important for patients and following treatments. In this study, a preliminary study that identifies MS from healthy controls with the help of magnetic resonance imaging (MRI) was investigated and implemented.

Recently, researchers tend to use computer vision and image processing (Zhang and Wu, 2008, 2009; Zhang et al., 2009a,b, 2010a,b) techniques to accomplish MS automatic-identification tasks. For instances, Murray et al. (2010) proposed to use multiscale amplitude modulation and frequency modulation (AM-FM) to identify MS. Nayak et al. (2016) presented a novel method, combining AdaBoost with random forest (ARF). Wang et al. (2016) combined biorthogonal wavelet transform (BWT) and logistic regression (LR). Wu and Lopez (2017) used four-level Haar wavelet transform (HWT). Zhang et al. (2017) proposed a novel MS identification system based on Minkowski-Bouligand Dimension (MBD).

Above methods secured promising results. Nevertheless, their methods need to extract features beforehand, and they need to validate their hand-extracted features effective (Chang, 2018a,b,c; Lee et al., 2018). Recently, convolutional neural network (CNN) attracts the research interest of scholars, since it can mechanically develop the features by its early layers. CNN has already been applied to many fields, such as biometric identification (Das et al., 2019), manipulation detection (Bayar and Stamm, 2018), etc. Zhang et al. (2018) is the first to apply CNN to identify MS, and their method achieved an overall accuracy of 98.23%.

This study is based on the CNN structure of Zhang et al. (2018). We proposed two other improvements: batch normalization and stochastic pooling. In addition, we used dynamic learning rate to accelerate the convergence. Learning rate is a parameter to control how quickly the proposed model converge to a local minimal. Low learning rate means a slow speed toward the downward slope. However, it can certain that we won't miss the local minimum but a long time to converge. Therefore, in our research, we set the learning rate a large value and reduce it by every given number of epochs instead of the fixed small learning rate until achieve convergence.

The rest of this paper is organized as follows: section Data Preprocessing described the data processing including data sources and data preprocessing. Section Methodology illustrates the method used in our research. Section Experiments, Results, and Discussions provided the experiment result and discussion.

## Data preprocessing

### Two sources

The dataset in this study were obtained from Zhang et al. (2018). First, MS images were obtained from the eHealth laboratory (2018). All brain lesions were identified and delineated by experienced MS neurologists, and were confirmed by radiologists. Second, the healthy controls were used from 681 slices of 26 healthy controls provided in Zhang et al. (2018). Table 1 shows the demographic characteristics of two datasets.

**Table 1:** Demographic characteristics of two datasets.

**Dataset**	**Source**	**# Subjects**	**Number of Slice**	**Age**	**Gender (m/f)**
Multiple sclerosis (2018)	eHealth	38	676	34.1 ± 10.5	17/21
Healthy control (Zhang et al., 2018)	private	26	681	33.5 ± 8.3	12/14

**Figure 1:**
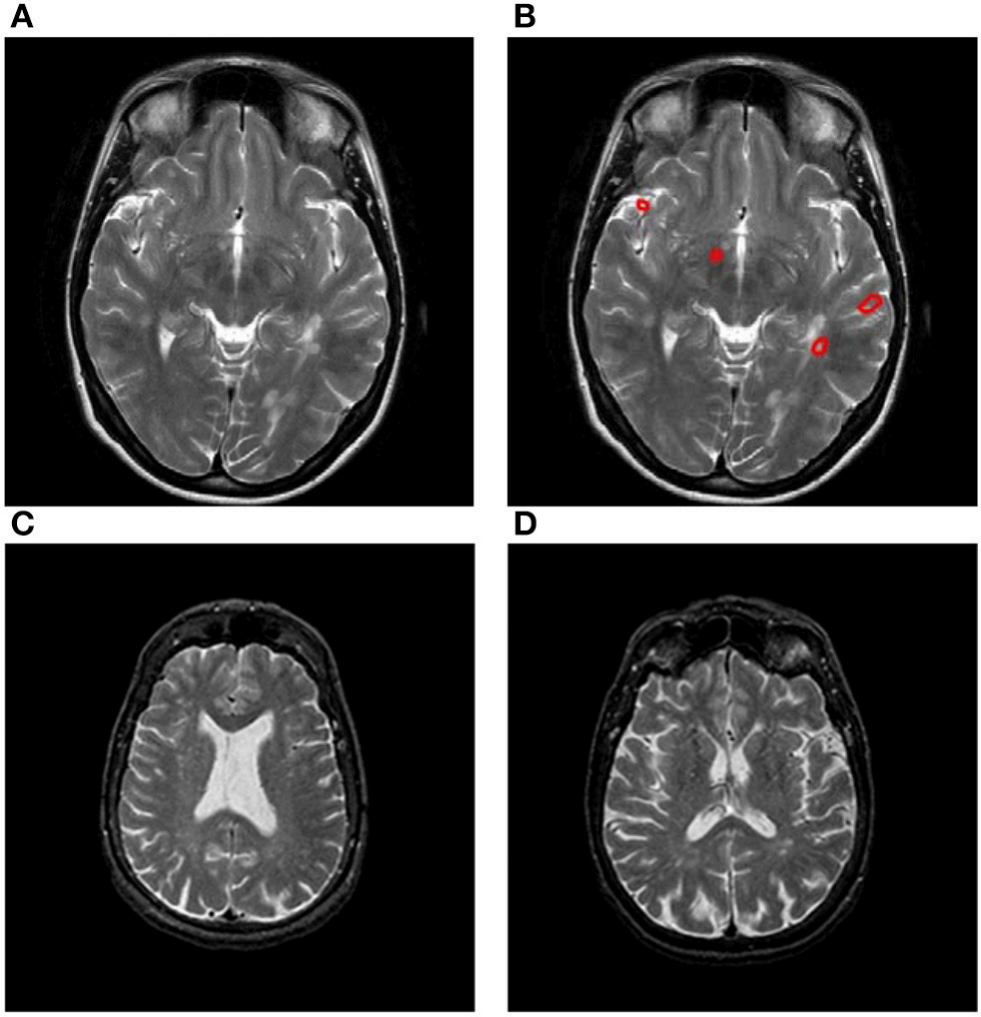
Samples of our dataset. **(A)** Original MS image. **(B)** MS image with plaque delineated. **(C)** Healthy control image I. **(D)** Healthy control image II.

Figure 1A shows the original slice, and Figure 1B shows the delineated results with four plaques, Areas surrounded by red line denotes the plaque. Figures 1C,D presents two slices from healthy controls.

### Contrast normalization

The brain slices are from two different sources; hence, the scanner machines may have different hardware setting (scanning sequence) and software settings (reconstruction from k-space, the store format, etc.). It is necessary to match the two sources of images in terms of gray-level intensities. This is also called contrast normalization, with aim of achieving consistency in dynamic range of various sources of data.

Histogram stretching (HS) method (Li et al., 2018) was chosen due to ease of implementation. HS aims to enhance the contrast by stretching the range of intensity values of two sources of images to the same range, providing the effect of inter-scan normalization.

The contrast normalization is implemented in following way. Let us assume μ is the original brain image, and φ is the contrast-normalized image, the process of HS can be described as

(1) $$\begin{array}{r} \varphi \left(x,y\right)=\frac{\mu \left(x,y\right)-\mu_{\min}}{\mu_{\max}-\mu_{\min}} \end{array}$$

where (*x, y*) represents the coordinate of pixel, μ_min_ and μ_max_ represents the minimum and maximum intensity values of original brain image μ.

(2) $$\begin{array}{r} \mu_{\min}=\underset{x}{\min}\underset{y}{\min}\left(\mu \left(x,y\right)\right) \end{array}$$

(3) $$\begin{array}{r} \mu_{\max}=\underset{x}{\max}\underset{y}{\max}\left(\mu \left(x,y\right)\right) \end{array}$$

We do contrast normalization for both two data of different sources, and finally combine them together, forming a 676+681 = 1,357-image dataset.

## Methodology

**Figure 2:**
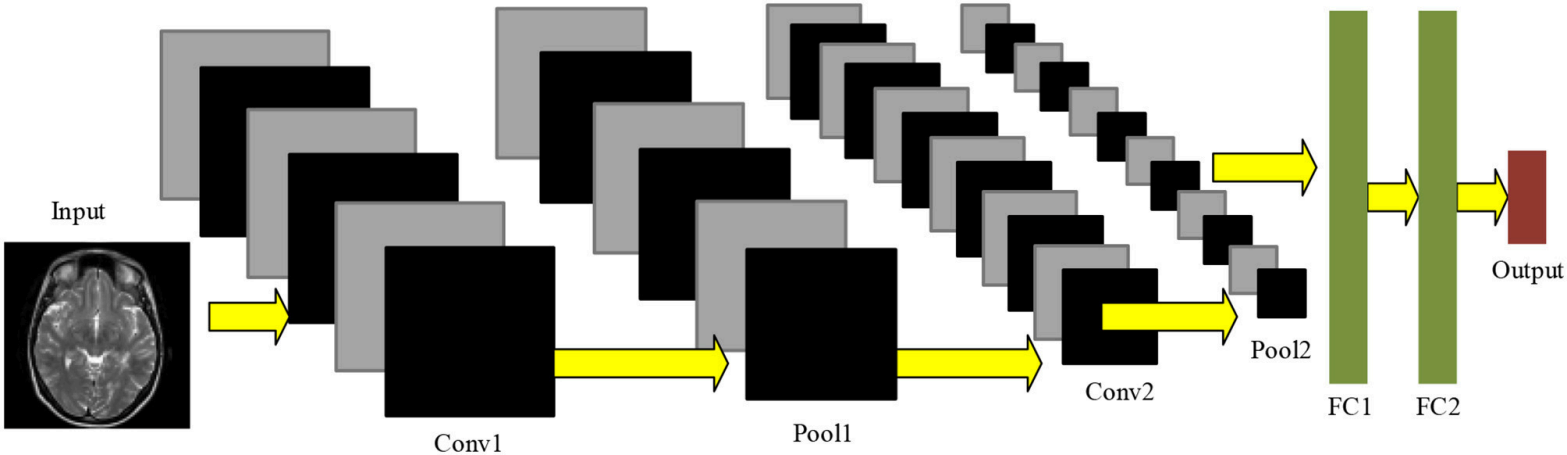
Pipeline of convolutional neural network.

Convolutional neural network is usually composed of conv layers, pooling layer, and fully connected layers. Figure 2 gives a toy example that consists of two conv layers, two pooling layers, and two fully connected layers. CNN can achieve comparable or even better performance than traditional AI approaches, while it does not need to manual design the features (Zeng et al., 2014, 2016a,b, 2017a,b).

### Conv layer

**Figure 3:**
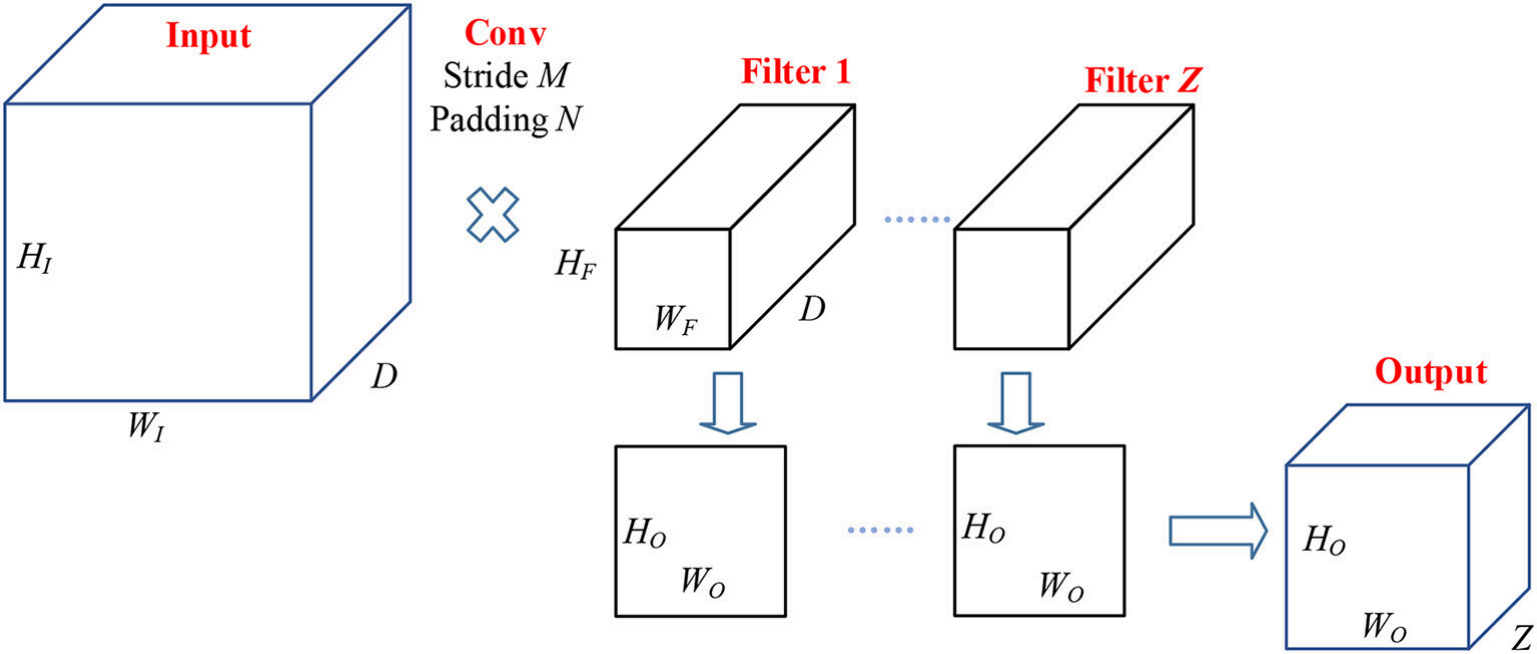
Pipeline of conv layer.

The conv layers performed Two-dimensional convolution along the width and height directions (Yu et al., 2018). It is worth noting that the weights in CNN are learned from backpropagation, except for initialization that weights are given randomly. Figure 3 shows the pipeline of data passing through a conv layer. Suppose there is an input with size of

(4) $$\begin{array}{r} \text{Input}:H_{I}\times W_{I}\times D \end{array}$$

where *H*_*I*_*, W*_*I*_, and *C* represent the height, width, and channels of the input, respectively.

Suppose the size of filter is

(5) $$\begin{array}{r} \begin{array}{c} \text{Filter 1}:H_{F}\times W_{F}\times D\\ \ldots \\ \text{Filter}Z:H_{F}\times W_{F}\times D \end{array} \end{array}$$

where *H*_*F*_ and *W*_*F*_ are height and width of each filter, and the channels of filter should be the same as that of the input. *Z* denotes the number of filters. Those filters move with stride of *M* and padding of *N*, then the channels of output activation map should be *Z*. The output size is:

(6) $$\begin{array}{r} \text{Output}:H_{O}\times W_{O}\times Z \end{array}$$

where *H*_*O*_ and *W*_*O*_ are the height and width of the output. Their values are:

(7) $$\begin{array}{r} H_{O}=1+\lfloor \frac{2N+H_{I}-H_{F}}{M}\rfloor \end{array}$$

(8) $$\begin{array}{r} W_{O}=1+\lfloor \frac{2N+W_{I}-W_{F}}{M}\rfloor \end{array}$$

where ⌊⌋ denotes the floor function. The outputs of conv layer are usually passed through a non-linear activation function, which normally chooses as rectified linear unit (ReLU) function.

### Pooling layer

The activation map contains too much features which can lead to overfitting and computational burden. Pooling layer is often used to implement dimension reduction. Furthermore, pooling can help to obtain invariance to translation. There are two commonly-used pooling methods: average pooling (AP), max pooling (MP).

The average pooling (Ibrahim et al., 2018) is to calculate the average value of the elements in each pooling region, while the max pooling is to select the max value of the pooling region. Suppose the region *R* contains pixelsχ, the average pooling and max pooling are defined as:

(9) $$\begin{array}{r} \text{AP:}\left\{\;y_{j}=\chi_{i}/\underset{i\in R_{j}}{\sum }\chi_{i}\right\} \end{array}$$

(10) $$\begin{array}{r} \text{MP:}\left\{\;y_{j}=\underset{i\in R_{j}}{\max}\chi_{i}\right\} \end{array}$$

**Figure 4:**
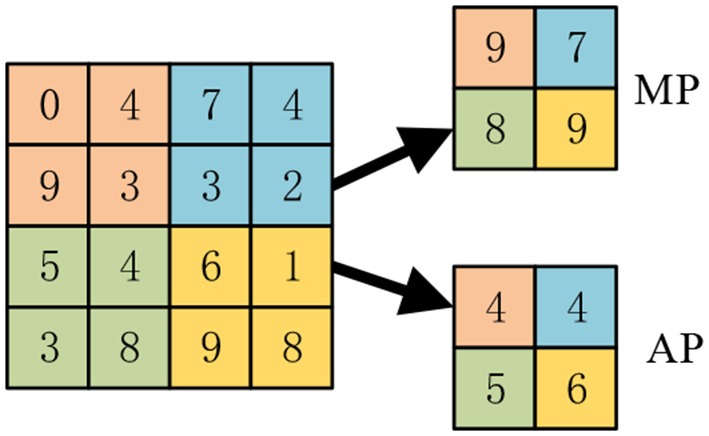
A toy example of max pooling and average pooling.

Figure 4 shows the difference, where the kernel size equals 2 and stride equals 2. The max pooling finally outputs the maximum values of all four quadrants, while the average pooling outputs the average values.

### Softmax and fully-connected layer

**Figure 5:**
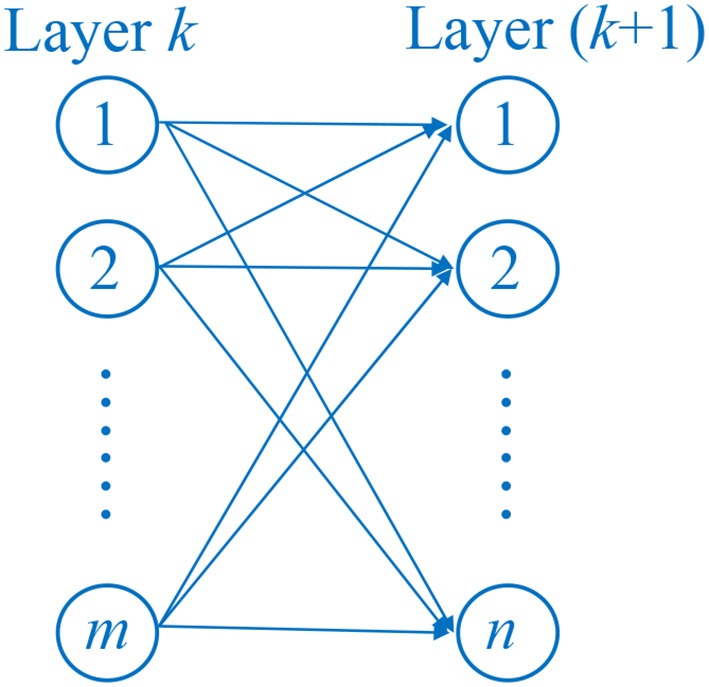
Structure of FC layer.

In fully connected (FC) layer, each neuron connects to all neurons of the previous layer, which makes this layer produce many parameters in this layer. The fully connected layer multiplied the input by a weight matrix and added to a bias vector. Suppose layer *k* contains *m* neurons, layer (*k*+1) contains *n* neurons. The weight matrix will be of size of *m* × *n*, and the bias vector will be size of 1 × *n*. Figure 5 shows the structure of FC layer.

Meanwhile, fully connected layer is often followed by a softmax function used to convert the input to a probability distribution. Here the “softmax” in this study only denotes the softmax function. While some literature will add a fully-connected layer before the softmax function and call the both layers as “softmax function.”

### Dropout

Deep neural network provides strong learning ability even for very complex function which is hard to understand by human. However, one problem often happened during the training of the deep neural network is overfitting, which means the error based on the training set is very small, but the error is large when the test data is provided to the neural network. We name it as bad generation to new dataset.

**Figure 6:**
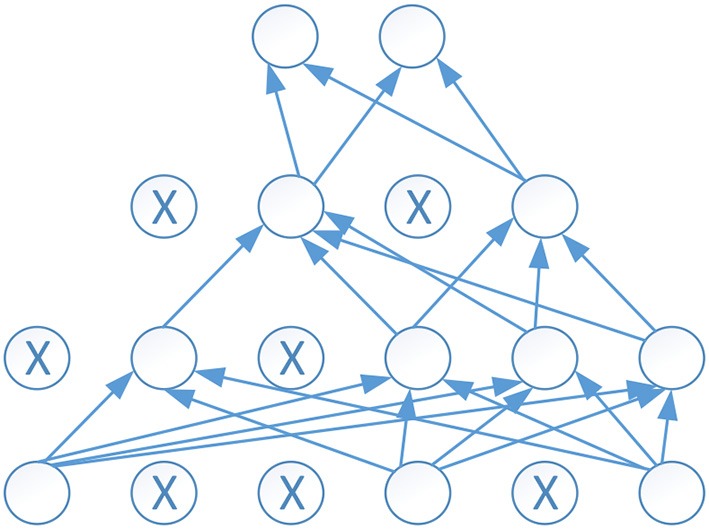
An example of dropout neural network.

Dropout was proposed to overcome the problem of overfitting. Dropout works as randomly set some neurons to zero in each forward pass. Each unit has a fixed probability *p* independent of the other units to be dropped out. The probability *p* is commonly set as 0.5. Figure 6 shows an example of dropout neural network, where the empty circle denotes a normal neuron, and a circle with X inside denotes a dropout neuron. It is obvious using dropout can reduce the links and make the neural network easy to train.

### Batch normalization

As the change of each layer's input distribution caused by the updating of the parameter in the previous layer, which is called as internal covariate shift, can result the slow training. Thus, to solve this problem, we employ the batch normalization to normalizes the layer's inputs over a mini batch to make the input layer have a uniform distribution. All the variables are listed in Table 2, then the batch normalization can be implemented as follows:

(11) $$\begin{array}{r} \alpha^{l}=\frac{1}{m}\underset{i}{\sum }z^{li} \end{array}$$

(12) $$\begin{array}{r} \sigma^{l2}=\frac{1}{m}{\underset{i}{\sum }\left(z^{li}-\alpha^{l}\right)}^{2} \end{array}$$

(13) $$\begin{array}{r} {\text{z}}_{norm}^{li}=\frac{{\text{z}}^{li}-\alpha^{l}}{\sqrt{\delta^{l2}+\varepsilon }} \end{array}$$

(14) $$\begin{array}{r} {\tilde{z}}^{li}\text{=}\lambda^{l}{\text{z}}_{norm}^{li}+\beta^{l} \end{array}$$

Here, ε is employed to improve numerical stability while the mini-batch variance is very small. Usually is set as default value *e*^−5^. However, the offset β and scale factor γ are updated during training as learnable parameters.

**Table 2:** Variables used in batch normalization.

**Parameter**	**Meaning**
*z*	The output of a layer
*z_*norm*_*	The normalization of *z*
~li	Input of the non-linearity layer
α	Mean value of the minibatch
δ^2^	Variance of the minibatch
*l*	Layer index
*i*	*i^*th*^* data in the mini batch
ε	A small constant
*m*	The number of samples of the minibatch

### Stochastic pooling

The stochastic pooling is proposed to overcome the problems caused by the max pooling and average pooling. The average pooling has a drawback, that all elements in the pooling region are considered, thus it may down-weight strong activation due to many near-zero elements. The max pooling solves this problem, but it easily overfits the training set. Hence, max pooling does not generalize well to test set.

Instead of calculating the mean value or the max value of each pooling region, the output of the stochastic pooling is obtained via sampling from a multinomial distribution formed from the activations of each pooling region *R*_*j*_. The procedure can be expressed as follows:

(1) Calculate the probability *p* of each element χ within the pooling region.

(15) $$\begin{array}{r} p_{i}=\frac{\chi_{i}}{\sum_{k\in R_{j}}\chi_{k}} \end{array}$$

in which, *k* is the index of the elements within the pooling region.

(2) Pick a location *l* within the pooling region according to the probability *p*. It is calculated by scanning the pooling region from left to right and up to bottom.

(16) $$\begin{array}{r} {\text{A}}_{j}=\chi_{l},l\sim P\left(p_{1},\ldots ,p_{\left|R_{j}\right|}\right) \end{array}$$

**Figure 7:**
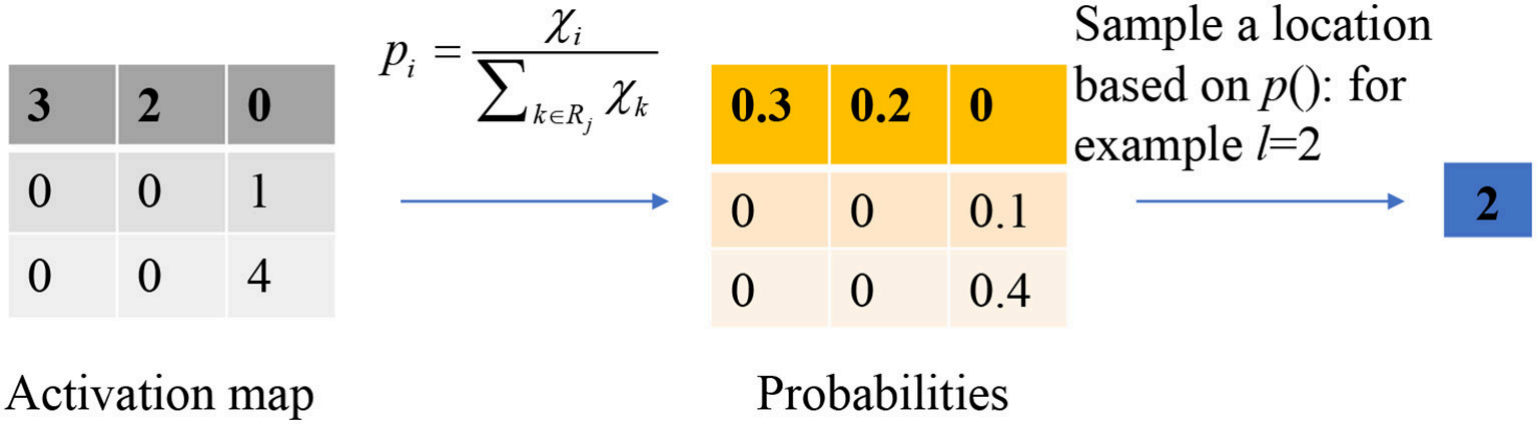
A toy example of stochastic pooling.

Instead of considering the max values only, stochastic pooling may use non-maximal activations within the pooling region. Figure 7 shows a toy example of using stochastic pooling. We first output the probabilities of the input matrix, then the roulette wheel falls within the pie of 0.2. Hence the location *l* is finally chosen as 2, and the output is the value at second position.

## Experiments, results, and discussions

### Division of the dataset

Hold-out validation method (Monteiro et al., 2016) was used to divide the dataset. In the training set, there are 350 MS images and 350 HC images. In the test set, we have 326 MS images and 331 HC images. Table 3 presents the setting hold-out validation method.

**Table 3:** Hold-out validation setting.

	**Training**	**Test**
MS	350	326
HC	350	331
Total	700	657

The dataset is divided into two parts without validation dataset for our research: training dataset and test dataset as shown in Table 3. The missing of validation set is mainly because of following reasons: First, according to the past research, validation set error rate may tend to overestimate the test error rate for the model fit on the entire data set (Bylander, 2002; Whiting et al., 2004). Second, as in order to avoid the overfitting, in addition of the training and test datasets, the validation dataset is necessary to tune the classification parameters. However, in this paper, we employed the drop out to overcome the problem of overfitting. The experiment result showed that there is no overfitting existing. Therefore, validation dataset is not used in our research.

### Data augmentation results

The deep learning usually needs a large amount of samples. However, ass it is a well-known challenge to collect biomedical data so as to generate more data from the limited data. Meanwhile, data augmentation has been shown to overcome the overfitting and increase the accuracy of classification tasks (Wong et al., 2016; Velasco et al., 2018). Therefore, in this study, we employed five different data augmentation (DA) methods to enlarge the training set (Velasco et al., 2018). First, we used image rotation. The rotation angle θ was set from −30 to 30° in step of 2°. The second DA method was scaling. The scaling factor*s* varied from 0.7 to 1.3 with step of 0.02. The third DA method was noise injection. The zero-mean Gaussian noise with variance of 0.01 was added to the original image to generate 30 new noise-contaminated images due to the random seed. The fourth DA method used was random translation by 30 times for each original image. The value of random translation *t* falls within the range of [0, 15] pixels, and obeys uniform distribution. The fifth DA method was gamma correction. The gamma-value *r* varied from 0.4 to 1.6 with step of 0.04.

**Figure 8:**
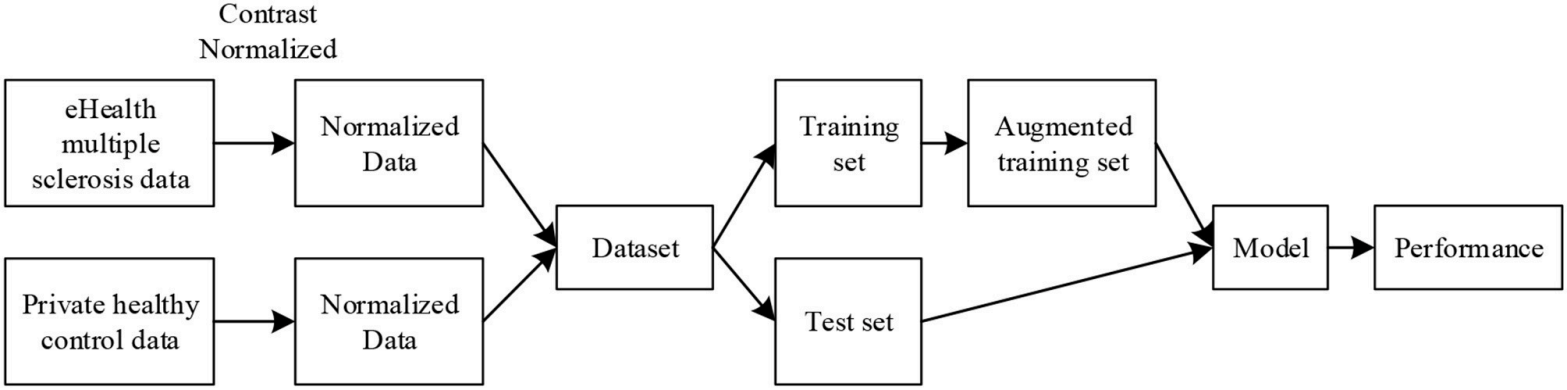
Pipeline of data preprocessing.

**Figure 9:**
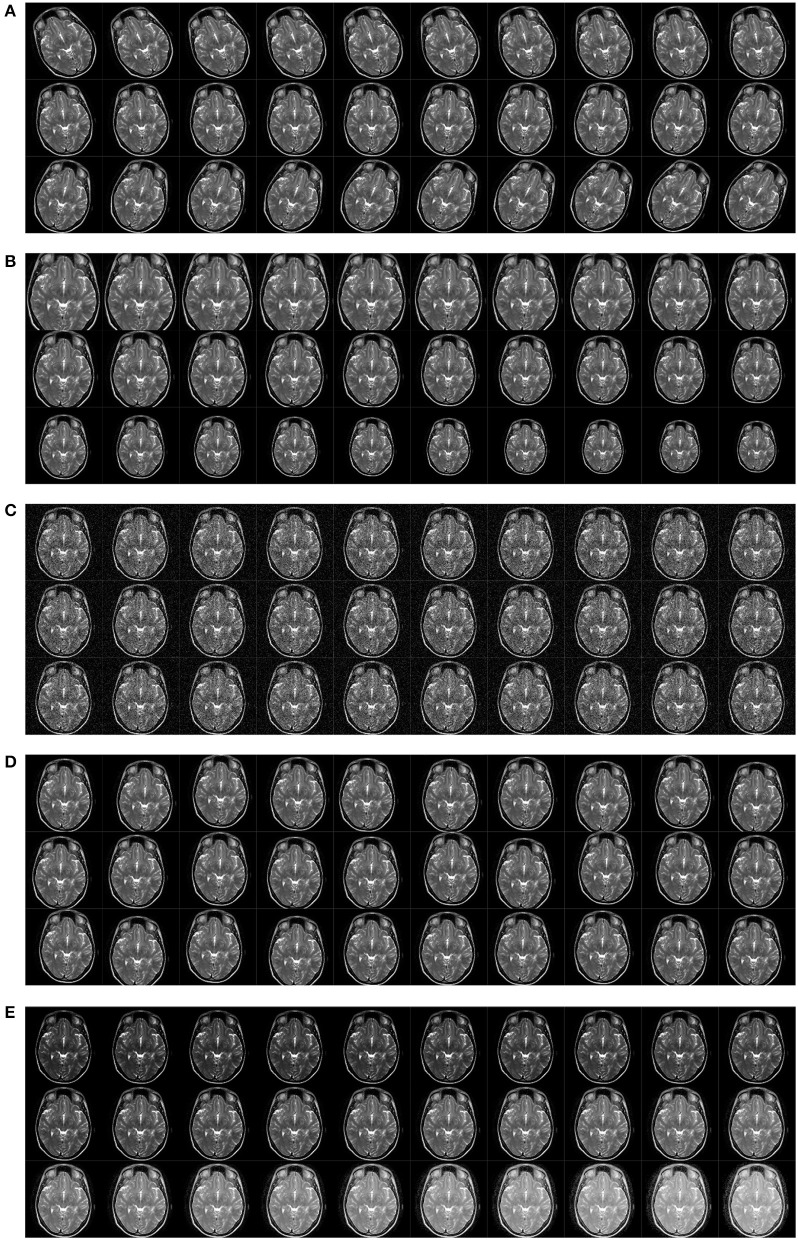
Results of data augmentation. **(A)** Rotation. **(B)** Scaling. **(C)** Noise injection. **(D)** Random translation. **(E)** Gamma correction.

The original training is presented in Figures 1A, 8 shows the pipeline of the data preprocessing, where the augmented training set is used to create a deep convolutional neural network model, and this trained model was tested over the test set, with final performance reported in Table 6. Figure 9A shows the results of image rotation. Figure 9B shows the image scaling results. Figures 9C–E shows the results of noise injection, random translation, and Gamma correction, respectively. As is shown, one training image can generate 150 new images, and thus, the data-augmented training image set is now 151x size of original training set.

### Structure of proposed CNN

**Figure 10:**
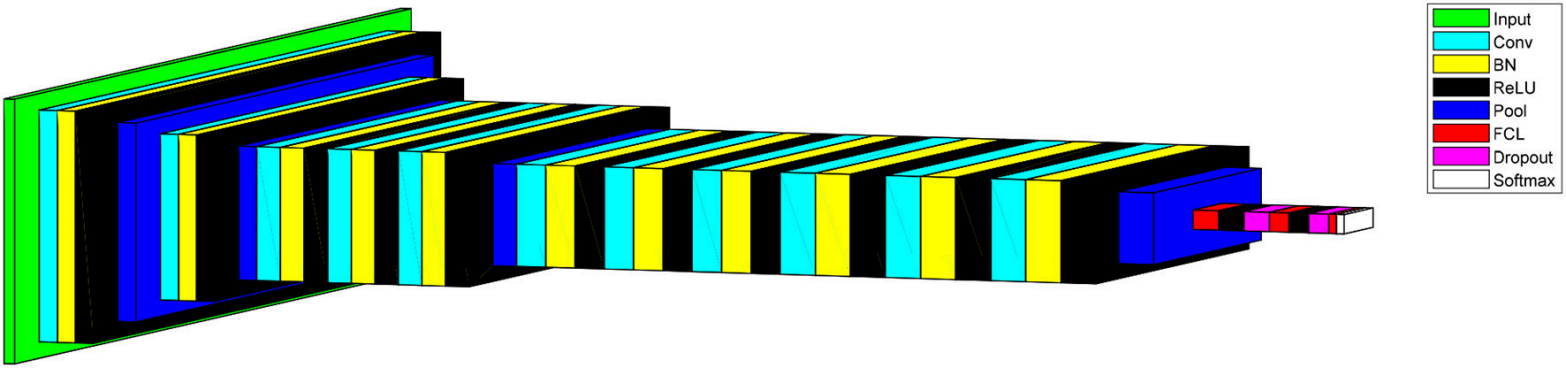
Activation map of proposed CNN model.

We built a 14-layer CNN model, with 11 conv layers and 3 fully-connected layers. Here we did not the number of other layers as convention. The hyperparameters were fine-tuned and their values were listed in Tables 4, 5. The padding values of all layers are set as “same.” Figure 10 shows the activation map of each layer. It is obvious that the height of width of output of each layer shrinks as going to the late layers.

**Table 4:** Hyperparameters of Conv layers.

**Layer**	**Filter size**	**# Channel**	**# Filters**	**Stride**
Conv_1	3 × 3	1	8	2
Pool_1	3 × 3			2
Conv_2	3 × 3	8	8	2
Pool_2	3 × 3			2
Conv_3	3 × 3	8	16	1
Conv_4	3 × 3	16	16	1
Conv_5	3 × 3	16	16	1
Pool_3	3 × 3			2
Conv_6	3 × 3	16	32	1
Conv_7	3 × 3	32	32	1
Conv_8	3 × 3	32	32	1
Conv_9	3 × 3	32	64	1
Conv_10	3 × 3	64	64	1
Conv_11	3 × 3	64	64	1
Pool_4	3 × 3			2

**Table 5:** Hyperparameters of Fully-connected layers.

**Layer**	**Weights**	**Bias**	**Probability**
FCL_1	20 × 1024	20 × 1
DO_1			0.5
FCL_2	10 × 20	10 × 1
DO_2			0.5
FCL_3	2 × 10	2 × 1

### Statistical results

**Figure 11:**
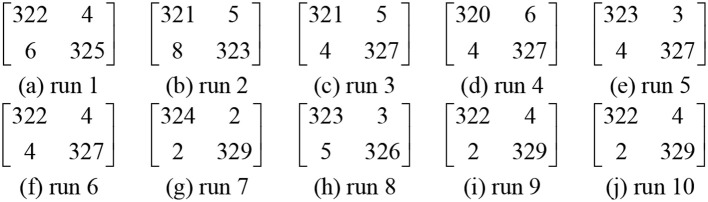
Confusion matrixes of each run.

We used our 14-layer CNN with “DO-BN-SP.” We ran the test 10 times, each time the hold-out division was updated randomly. The results over 10 runs are shown in Table 6. The average of sensitivity, specificity, and accuracy are 98.77 ± 0.35, 98.76 ± 0.58, and 98.77 ± 0.39, respectively. The confusion matrix of all runs are listed in Figure 11.

**Table 6:** Statistical analysis of 10 runs.

**Run**	**Sensitivity**	**Specificity**	**Precision**	**Accuracy**
1	98.77	98.19	98.17	98.48
2	98.47	97.58	97.57	98.02
3	98.47	98.79	98.77	98.63
4	98.16	98.79	98.77	98.48
5	99.08	98.79	98.78	98.93
6	98.77	98.79	98.77	98.78
7	99.39	99.40	99.39	99.39
8	99.08	98.49	98.48	98.78
9	98.77	99.40	99.38	99.09
10	98.77	99.40	99.38	99.09
Average	98.77 ± 0.35	98.76 ± 0.58	98.75 ± 0.58	98.77 ± 0.39

### Pooling method comparison

In this experiment, we compared the stochastic pooling (SP) with max pooling (MP) and average pooling (AP). All the other settings are fixed and unchanged. The results of 10 runs of MP and AP are shown in Table 7.

**Table 7:** Ten random runs of MP and AP methods.

	**Sensitivity (%)**	**Specificity (%)**	**Precision (%)**	**Accuracy (%)**
**MP**				
R1	97.87	97.87	97.89	97.87
R2	98.63	98.63	98.66	98.63
R3	98.18	98.18	98.20	98.17
R4	96.04	96.04	96.11	96.04
R5	96.80	96.80	96.86	96.80
R6	98.78	98.78	98.81	98.78
R7	98.63	98.63	98.65	98.63
R8	97.86	97.86	97.88	97.87
R9	99.24	99.24	99.25	99.24
R10	98.63	98.63	98.64	98.63
Average	98.07 ± 0.93	98.07 ± 0.98	98.10 ± 0.96	98.07 ± 0.98
**AP**				
1	97.41	97.41	97.55	97.41
2	96.65	96.66	96.67	96.65
3	98.33	98.32	98.37	98.33
4	97.41	97.41	97.42	97.41
5	96.65	96.65	96.65	96.65
6	97.87	97.87	97.88	97.87
7	97.56	97.57	97.58	97.56
8	97.87	97.87	97.92	97.87
9	98.48	98.48	98.52	98.48
10	98.48	98.47	98.51	98.48
Average	97.67 ± 0.64	97.67 ± 0.67	97.71 ± 0.68	97.67 ± 0.67

We performed Wilcoxon signed rank test (Keyhanmehr et al., 2018) between the results of SP and those of MP, and between the results of SP and those of AP. The results are listed in Table 8. It shows SP are significantly better than MP in terms of specificity and accuracy. Meanwhile, SP are significantly better than AP in all four measures.

**Table 8:** Pooling method comparison and *p*-values of singed-rank test.

**Pooling**	**Sensitivity (%)**	**Specificity (%)**	**Precision (%)**	**Accuracy (%)**
MP	98.33 ± 0.75	98.33 ± 0.79	98.34 ± 0.79	98.33 ± 0.80
*p*-value (SP-MP)	0.0645	**0.0469**	0.0605	**0.0430**
AP	97.67 ± 0.64	97.67 ± 0.67	97.71 ± 0.68	97.67 ± 0.67
*p*-value (SP-AP)	**0.0020**	**0.0020**	**0.0020**	**0.0020**
SP (Ours)	98.77 ± 0.35	98.76 ± 0.58	98.75 ± 0.58	98.77 ± 0.39

*Bold means the p-values are less than 0.05*.

In this section, Wilcoxon signed rank test was utilized instead of two-sample *t*-test (Jafari and Ansari-Pour, 2018) and chi-square test (Kurt et al., 2019) based on following reasons: two-sample *t*-test supposes the data comes from independent random samples of normal distributions, the same for chi-square goodness-of-fit test. However, our sensitivity/specificity/precision/accuracy data do not meet the condition of gaussian distribution.

### Validation of the data augmentation

We compared the training process with and without data augmentation to explore the augmentation strategies. The data augmentation methods including: image rotation, scaling, noise injection, random translation and gamma correction as stated in section Data Augmentation Results. The respective performance is shown in Table 9. Training with data augmentation could provide better performance, particularly reducing the range of standard deviation.

**Table 9:** Comparison of the approach with and without data augmentation.

**Approach**	**Sensitivity (%)**	**Specificity (%)**	**Precision (%)**	**Accuracy (%)**
No augmentation	98.22 ± 0.71	98.19 ± 1.03	98.18 ± 1.01	98.20 ± 0.77
Data augmentation	98.77 ± 0.35	98.76 ± 0.58	98.75 ± 0.58	98.77 ± 0.39

### Comparison to state-of-the-art approaches

**Figure 12:**
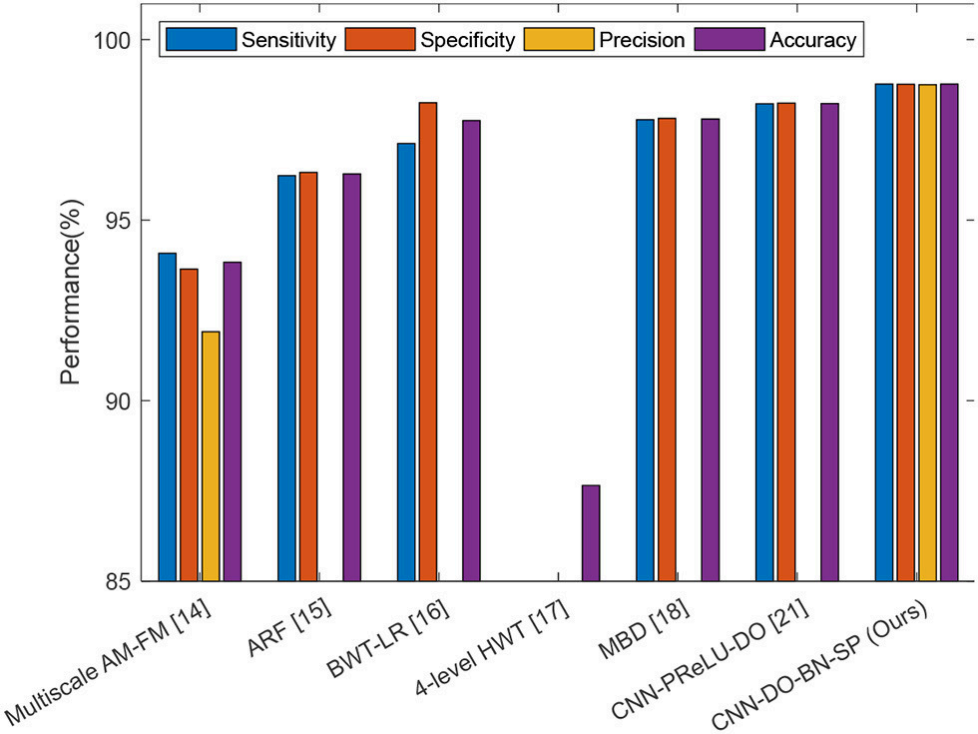
Comparison plot.

In this experiment, we compared our CNN-DO-BN-SP method with traditional AI methods: Multiscale AM-FM (Murray et al., 2010), ARF (Nayak et al., 2016), BWT-LR (Wang et al., 2016), 4-level HWT (Wu and Lopez, 2017), and MBD (Zhang et al., 2017). The results were presented in Table 10. Besides, we compared our method with a modern CNN method, viz., CNN-PReLU-DO (Zhang et al., 2018). The results were listed in Table 11. We can observe that our method achieved superior performance than all six state-of-the-art approaches, as shown in Figure 12.

**Table 10:** Comparison to traditional AI approaches.

**Approach**	**Sensitivity (%)**	**Specificity (%)**	**Precision(%)**	**Accuracy (%)**
Multiscale AM-FM (Murray et al., 2010)	94.08	93.64	91.91	93.83
ARF (Nayak et al., 2016)	96.23 ± 1.18	96.32 ± 1.48	N/A	96.28 ± 1.25
BWT-LR (Wang et al., 2016)	97.12 ± 0.14	98.25 ± 0.16	N/A	97.76 ± 0.10
4-level HWT (Wu and Lopez, 2017)	N/A	N/A	N/A	87.65 ± 1.79
MBD (Zhang et al., 2017)	97.78 ± 1.29	97.82 ± 1.60	N/A	97.80 ± 1.40
CNN-DO-BN-SP (Ours)	98.77 ± 0.35	98.76 ± 0.58	98.75 ± 0.58	98.77 ± 0.39

**Table 11:** Comparison to deep learning approaches.

**Approach**	**Sensitivity (%)**	**Specificity (%)**	**Precision (%)**	**Accuracy (%)**
CNN-PReLU-DO (Zhang et al., 2018)	98.22	98.24	N/A	98.23
CNN-DO-BN-SP (Ours)	98.77 ± 0.35	98.76 ± 0.58	98.75 ± 0.58	98.77 ± 0.39

The reason why our method is the best among all seven algorithms lies in four points. (i) We used data augmentation, to enhance the generality of our deep neural network. (ii) The batch normalization technique was used to resolve the internal covariate shift problem. (iii) Dropout technique was used to avoid overfitting in the fully connected layers. (iv) Stochastic pooling was employed to resolve the down-weight issue caused by average pooling and overfitting problem caused by max pooling.

The bioinspired-algorithm may help the design or initialization of our model. In the future, we shall try particle swarm optimization (PSO) (Zeng et al., 2016c,d) and other methods. The hardware of our model can be optimized using specific optimization method (Zeng et al., 2018).

In this paper, we employed data augmentation, the main benefits mainly as follows: As it is a well-know challenge to collect biomedical data so as to generate more data from the limited data. Second, data augmentation has been shown to overcome the overfitting and increase the accuracy of classification tasks (Wong et al., 2016; Velasco et al., 2018).

## Conclusion

In this study, we proposed a novel fourteen-layer convolutional neural network with three advanced techniques: dropout, batch normalization, and stochastic pooling. The main contributes are list as follows:

1. In this paper, we first applied CNN with stochastic pooling for the Multiple sclerosis detection whose early diagnosis is important for patients' following treatment.
2. In order to overcome the problems happened in the traditional CNN, such as the internal co shift invariant and overfitting, we utilized batch normalization and dropout.
3. Considering the size of the dataset, data augmentation was employed in our research for the train set.
4. The proposed method has the best performance compared to the other state of art methods in terms of sensitivity, specificity, precision and accuracy.

The results showed our method is superior to six state-of-the-art approaches: five traditional artificial intelligence methods and one deep learning method. The detail explanation is provided in section Comparison to State-of-the-art approaches. In the future, we shall try to test other pooling variants, such as pyramid pooling. The dense-connected convolutional networks will also be tested for our task. Meanwhile, we will also work on finding more ways to accelerate convergence (Liao et al., 2018).

## Author contributions

S-HW conceived the study. CT and JS designed the model. CT and Y-DZ analyzed the data. S-HW, PP, and Y-DZ acquired the preprocessed the data. JY and JS wrote the draft. CH, PP, and Y-DZ interpreted the results. All authors gave critical revision and consent for this submission.

### Conflict of interest statement

The authors declare that the research was conducted in the absence of any commercial or financial relationships that could be construed as a potential conflict of interest.
